# Biomechanical evaluation of position and bicortical fixation of anterior lateral vertebral screws in a porcine model

**DOI:** 10.1038/s41598-023-27433-6

**Published:** 2023-01-09

**Authors:** Ming-Kai Hsieh, De-Mei Lee, Yun-Da Li, Chun-Chin Peng, Tsung-Ting Tsai, Po-Liang Lai, Weng-Pin Chen, Ching-Lung Tai

**Affiliations:** 1 Department of Orthopaedic Surgery, Spine Section, Bone and Joint Research Center, Chang Gung Memorial Hospital, Taoyuan, Taiwan; 2 Department of Mechanical Engineering, Chang Gung University, Taoyuan, Taiwan; 3 Department of Biomedical Engineering, Chang Gung University, Taoyuan, Taiwan; 4 Department of Mechanical Engineering, National Taipei University of Technology, Taipei, Taiwan

**Keywords:** Orthopaedics, Bone

## Abstract

Although an anterior approach with anterior lateral screw fixation has been developed for stabilizing the thoracolumbar spine clinically, screw loosening still occurs. In this novel in vitro study, we attempted to elucidate the optimal screw position in the lateral lumbar vertebra and the effect of bicortical fixation. A total of 72 fresh-frozen lumbar vertebrae from L1–6 were harvested from 12 mature pigs and randomly assigned to two modalities: bicortical fixation (n = 36) and unicortical fixation (n = 36). Six groups of screw positions in the lateral vertebral body in each modality were designated as central-anterior, central-middle, central-posterior, lower-anterior, lower-middle, and lower- posterior; 6 specimens were used in each group. The correlations between screw fixation modalities, screw positions and axial pullout strength were analyzed. An appropriate screw trajectory and insertional depth were confirmed using axial and sagittal X-ray imaging prior to pullout testing. In both bicortical and unicortical fixation modalities, the screw pullout force was significantly higher in the posterior or middle position than in the anterior position (*p* < 0.05), and there was no significant differences between the central and lower positions. The maximal pullout forces from the same screw positions in unicortical fixation modalities were all significantly lower, decreases that ranged from 32.7 to 74%, than those in bicortical fixation modalities. Our study using porcine vertebrae showed that screws in the middle or posterior position of the lateral vertebral body had a higher pullout performance than those in the anterior position. Posteriorly positioned lateral vertebral screws with unicortical fixation provided better stability than anteriorly positioned screws with bicortical fixation.

## Introduction

Posterior pedicle screw fixation for spinal fusion surgery is a useful procedure for the treatment of spinal disorders, including fracture, degeneration, idiopathic scoliosis, infection or metastatic spinal tumors^1–5^. However, for large vertebral defects, the anterior thoraco-abdominal approach with spinal instrumentation anterior to the lesion has been proven to be more effective in immediate postoperative stability, deformity correction and neurological safety while eradicating the spinal lesion^6–11^. In recent years, oblique lumbar interbody fusion (OLIF) with supplemental anterior lateral screws-rod fixation has gained popularity in the context of degenerative lumbar disease^12,13^. This approach offers the advantage of direct visualization of the anterior and middle column pathology and allows anterior large support to be placed without irritating the nerve and avoids posterior pedicle screws fixation^10^. Several anterior fixation systems have been developed for stabilizing the thoracolumbar spine clinically, but screw loosening still occurs^14–17^. The anterior plating system has been widely compared with other biomechanical stabilization systems by using functional spinal units of human or swine cadavers^18–23^, but the importance of screw position on the lateral side of the vertebra and the effect of bicortical fixation have never been evaluated. The bone in the region between the screw thread crests is damaged and subsequently lowered the instrumented construct stability when a screw is pullout^24^. The individual screw axial pullout test has been proved to be correlated with construct stability^25,26^. In this novel in vitro experimental study, we compared the pullout strength of screws in six different positions relative to the lateral side of normal-density porcine vertebrae: central-anterior (C-a), central-middle (C-m), central- posterior (C-p), lower-anterior (L-a), lower-middle (L-m), and lower-posterior (L-p). Meanwhile, the present study is the first to conduct a biomechanical comparison between bicortical and unicortical fixation of anterior lateral vertebral screws.

## Results

**Figure 1:**
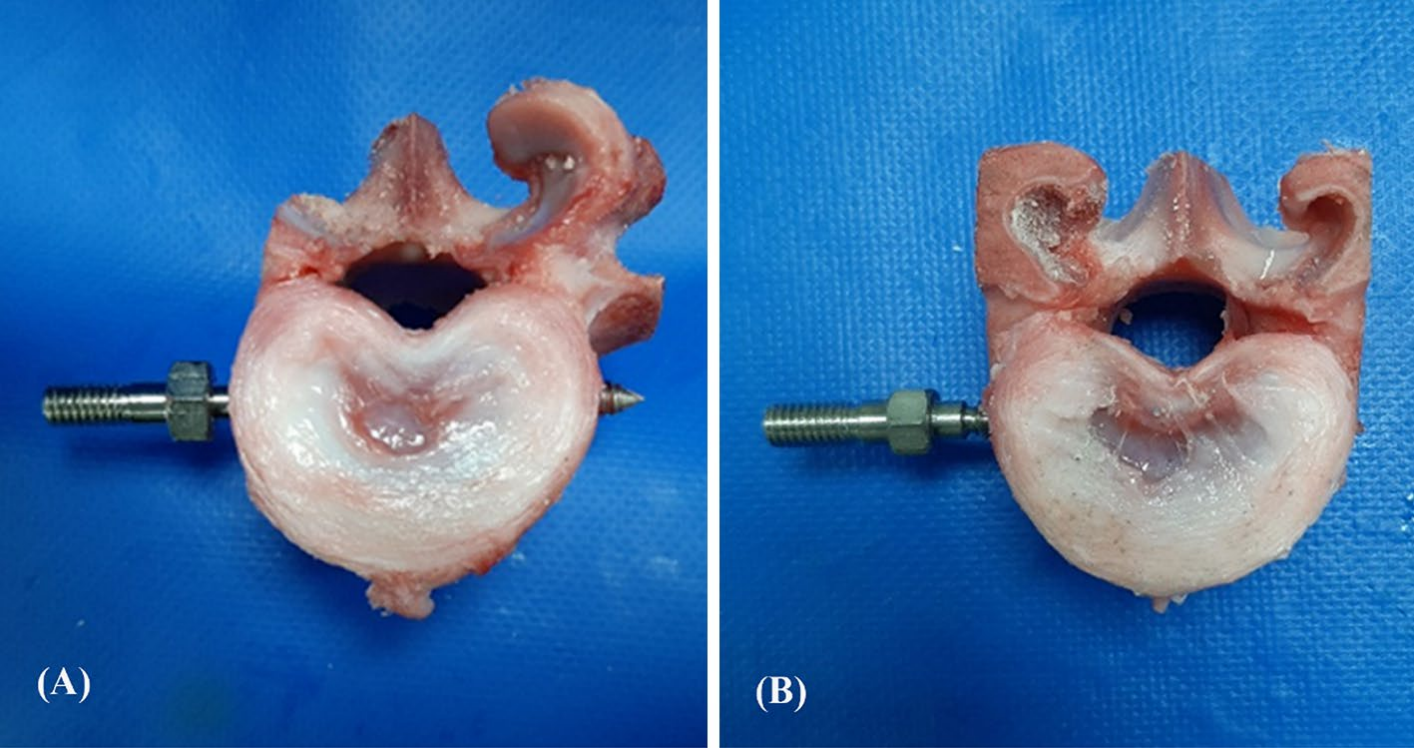
Photographs showing the axial view of the porcine vertebrae with bicortical and unicortical fixation. (**A**) At least 2 thread widths penetrated out of the opposite cortex in the bicortical fixation modality; and (**B**) no penetration was observed in the unicortical fixation modality.

**Figure 2:**
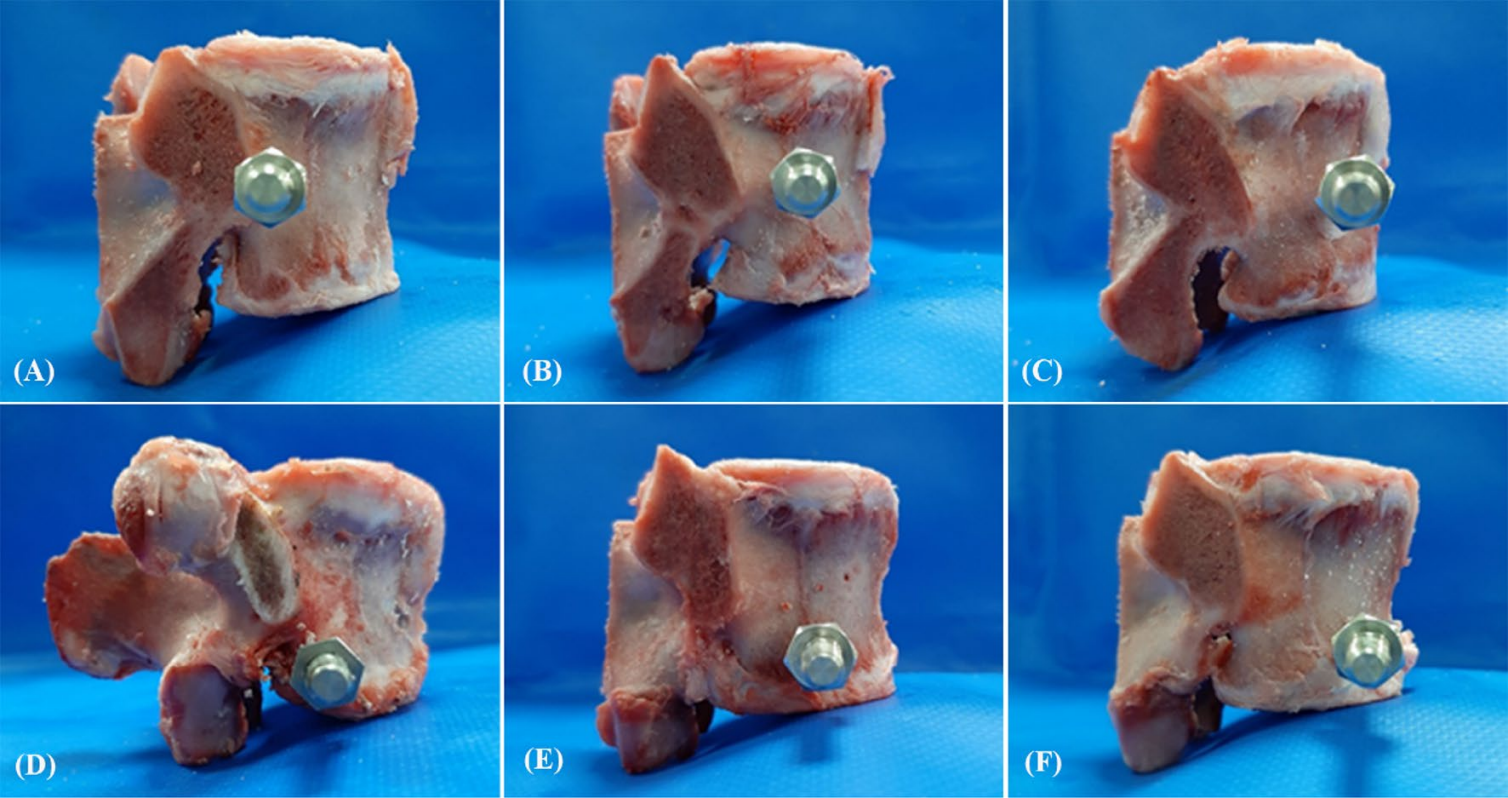
Photographs showing the sagittal view of the porcine vertebrae with various screw insertion positions. (**A**) C-p, (**B**) C-m, (**C**) C-a, (**D**) L-p, (**E**) L-m, and (**F**) L-a.

Before image analysis, no malposition or fractures in the implanted specimens were grossly observed (Figs. 1, 2). The top view showed the difference between two fixation modalities (Fig. 1) and the sagittal view showed six proper positions on lateral vertebral bodies (Fig. 2).

### X-ray image analysis

**Figure 3:**
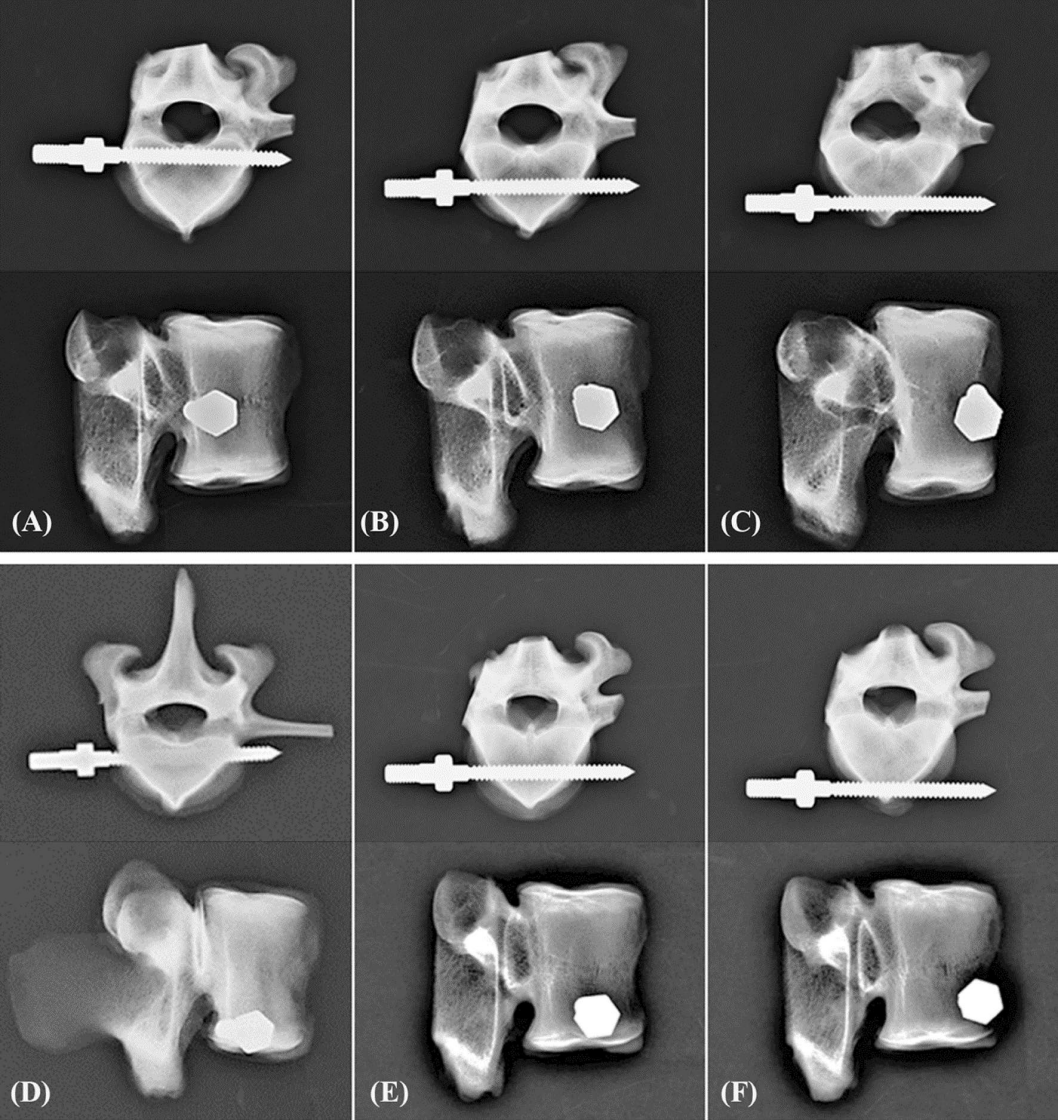
Axial (upper) and sagittal (bottom) X-ray images of the bicortical fixation modality showing the porcine vertebrae with various screw insertion positions. (**A**) C-p, (**B**) C-m, (**C**) C-a, (**D**) L-p, (**E**) L-m, and (**F**) L-a.

**Figure 4:**
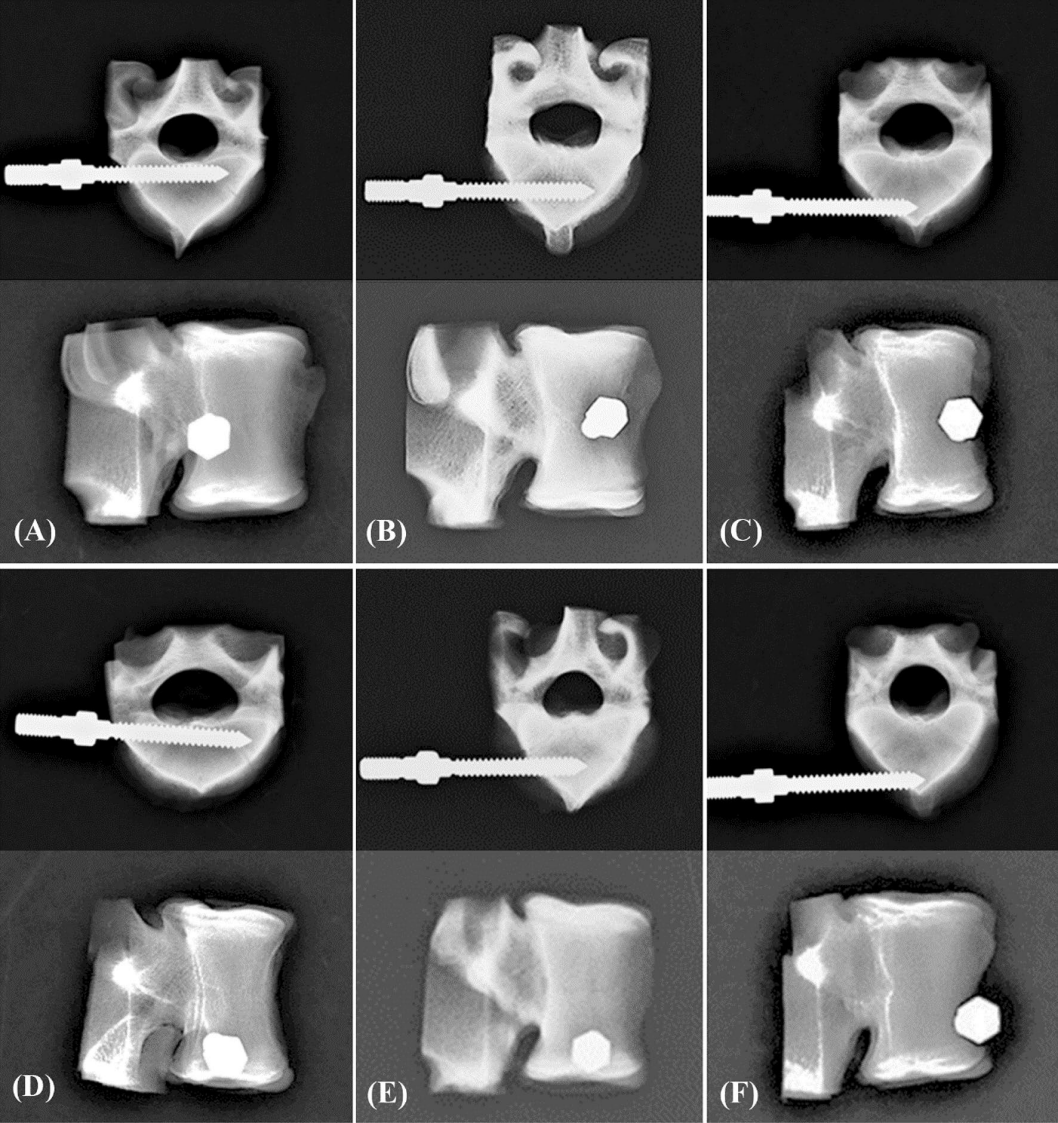
Axial (upper) and sagittal (bottom) X-ray images of the unicortical fixation modality showing the porcine vertebrae with various insertion positions. (**A**) C-p, (**B**) C-m, (**C**) C-a, (**D**) L-p, (**E**) L-m, and (**F**) L-a.

An appropriate screw trajectory and insertional depth were also confirmed using axial and sagittal X-ray imaging prior to pullout testing in the bicortical (Fig. 3) and unicortical fixation modalities (Fig. 4). All screws were parallel to the posterior cortex in the axial images (Figs. 3, 4, upper), and all screws were inserted into the proper of C-p, C-m, C-a, L-p, L-m, and L-a positions (Figs. 3, 4, bottom). For the bicortical fixation modality, at least 2 thread widths penetrated out of the opposite cortex (Fig. 3A–F, upper), whereas, there was at least 2 mm from the screw tip to the opposite cortex in the unicortical fixation modality (Fig. 4A–F, upper). No fractures or defects in the vertebrae were detected in either view.

### Pullout strength

**Figure 5:**
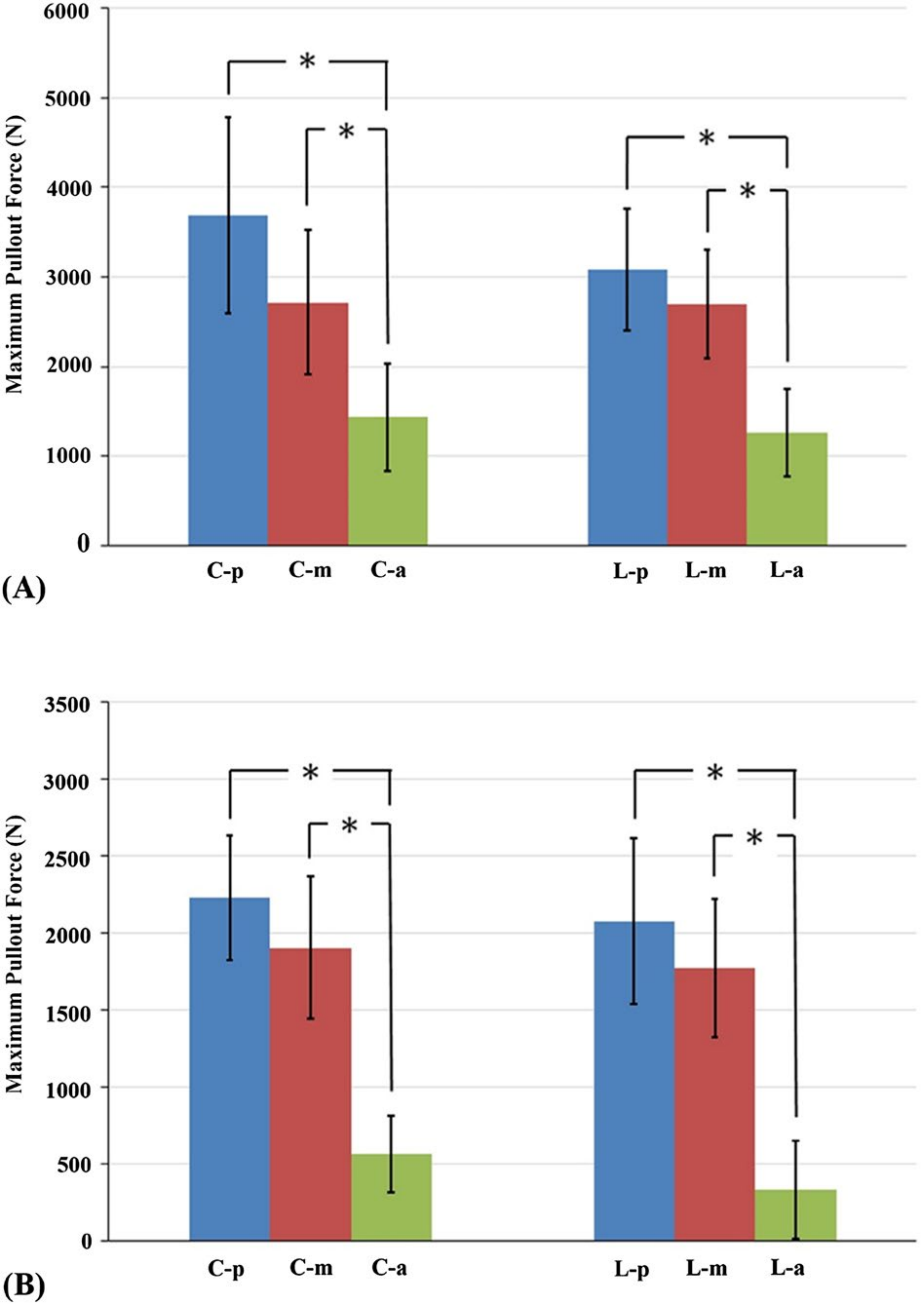
Mean maximum pullout forces for the (**A**) bicortical and (**B**) unicortical fixation modalities. For both bicortical and unicortical fixation modalities, the pullout force for the screws in the posterior or middle positions were significantly higher than that in the anterior position. There were no significant differences between the screws in the posterior and middle vertebrae positions and no difference between the screws in the central and lower vertebrae positions. Groups with statistically significant difference (p < 0.05) are indicated with “*” symbol.

**Figure 6:**
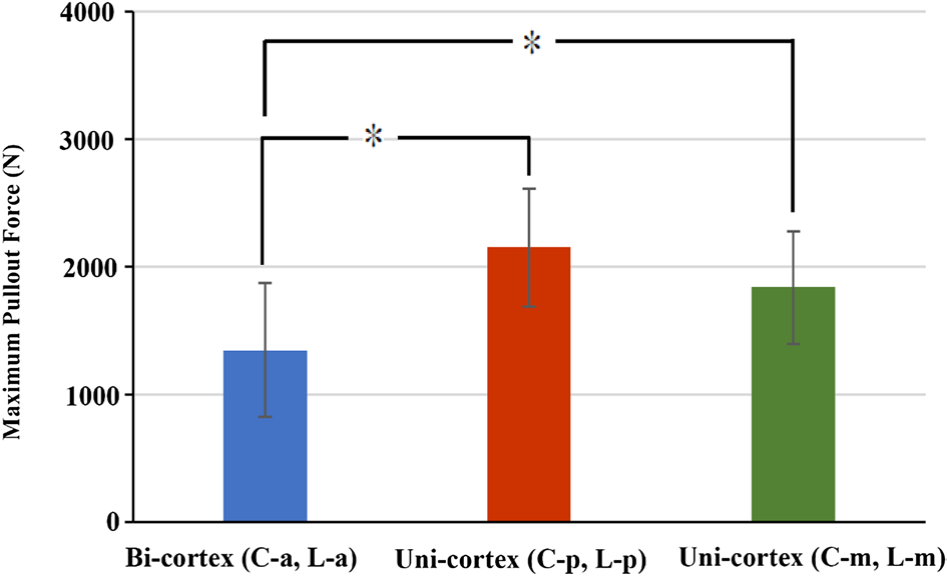
Mean maximum pullout forces for the bicortical purchased screws in anterior position and unicortical purchased screws in the posterior and middle position. Groups with statistically significant difference (p < 0.05) are indicated with “*” symbol.

**Figure 7:**
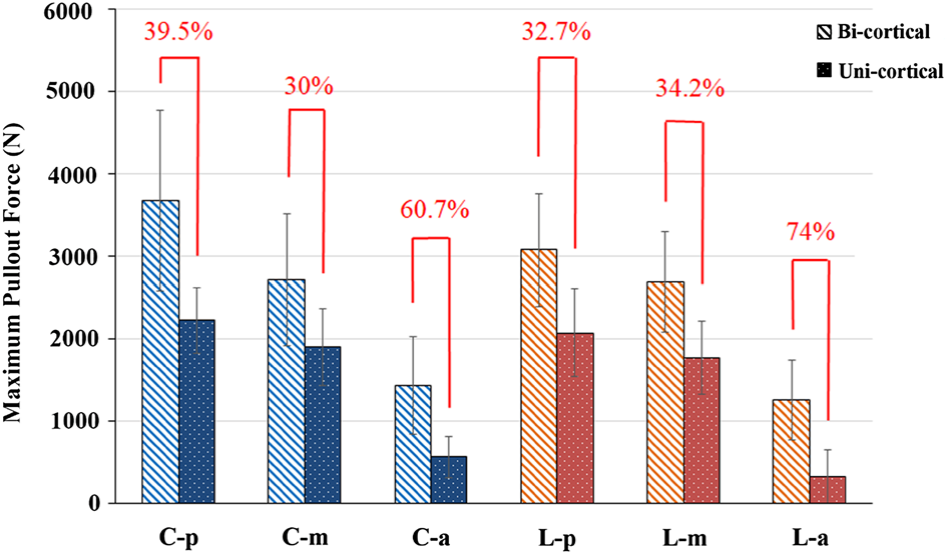
Comparison of maximum pullout forces between bicortical and unicortical fixation modalities.

In the bicortical fixation modalities, the C-p, C-m, C-a, L-p, L-m, and L-a groups had mean pullout force values of 3683.43 ± 1093.33 N, 2718.89 ± 802.31 N, 1434.16 ± 592.68 N, 3081.72 ± 682.25 N, 2692.62 ± 606.14 N, and 1261.61 ± 484.58 N, respectively (Fig. 5A). In the unicortical fixation modalities, the mean pullout force values in the C-p, C-m, C-a, L-p, L-m, and L-a groups were 2227.40 ± 403.09 N, 1902.87 ± 463.26 N, 563.94 ± 251.5 N, 2074.91 ± 537.65 N, 1772.26 ± 444.94 N, and 328.52 ± 319.28 N, respectively (Fig. 5B). For the same screw insertion location (C-p, C-m, C-a, L-p, L-m or L-a), bicortical fixation modality had a significant higher pullout force than that of unicortical fixation modality (p < 0.05). For the given modalities, pullout force of screws in posterior and middle position were significantly higher than that in the anterior position (p < 0.05) (Fig. 5). In Fig. 6, unicortical purchased screws in the posterior (2151.15 ± 459.98 N) and middle position (1837.57 ± 438.39 N) showed significant higher pullout force than bicortical purchased screws in anterior position (1347.89 ± 523.95 N) (p < 0.05). A comparison of pullout force between bicortical and unicortical fixation modalities in the various groups is shown in Fig. 7. The percentage of maximal pullout force in the unicortical fixation relative to the bicortical fixation were all significantly decreased, reaching to 32.7–74% for screws in the same position.

The values of the decreased pullout force percentage were 30–39.5% in the middle and posterior positions; however, much larger deceased values were reached, namely, 60.7–74%, when the screws were positioned over the anterior third of the vertebra (Fig. 7). There were no significant differences between the screws in the posterior and middle vertebrae positions and no difference between the screws in the central and lower vertebrae positions.

## Discussion

In both our bicortical and unicortical fixation modalities (Figs. 3, 4), the pullout force for the screws in the posterior and middle positions were significantly higher than that in the anterior position, and there was no significant difference between the central and lower positions. The pullout force for bicortical screws has been proved to be higher than that for unicortical screws in the dorsal–ventral direction of the proximal phalanx, proximal tibia and C1 lateral mass screw^27–29^. From a regional BMD analysis of 377 L3 vertebrae through quantitative micro CT, the central regions exhibited lower BMD than the peripheral regions^30^, which implies that the pullout force for bicortical fixation was expected to be higher than that for unicortical fixation in the vertebra. In our study (Fig. 10), the maximal pullout forces from the same screw position in the unicortical fixation modalities were all significantly lower than those in the bicortical fixation modalities. Trabecular bone in human vertebrae displays substantial heterogeneity in density and architecture throughout the vertebral body. Through quantitative micro CT and dual-energy X-ray absorptiometry (DEXA) scans, higher bone mineral density (BMD), bone volume and trabecular number were found in the posterior region than in the anterior region^30–33^. With respect to the superior–inferior axis, there are no differences across the superior, mid-transverse and inferior thirds^32,34^. Our biomechanical study is compatible with those radiographic results.

In an anatomical study with 40 adults of T9-L3 through *3D*-CT reconstruction, the average distance between the aorta and vertebral body ranged from 3.39 to 5.94 mm, and the distance between the inferior vena cava and vertebral body was even higher (5.74–10.77 mm)^35^. Both large vessels are in the anterior region, which eliminates the concern of vascular injury from bicortical fixation in the middle or posterior position. Interestingly, unicortical purchased screws in the posterior and middle position showed significant higher pullout force than bicortical purchased screws in anterior position (Fig. 6). This phenomenon suggested that posteriorly positioned lateral vertebral screws with unicortical fixation provided better stability without risking great vessel injury.

The availability of fresh frozen human cadaver is very limited and the porcine spine shared a more similar anatomical geometries with human compared to goats, sheep and dogs^36,37^ and study proved that similar biomechanical results between formalin fixated specimens with fresh frozen cadaver^38^. Iris Busscher et al.^39^ compared 16 anatomical dimensions from 6 human and 6 porcine spines through CT scans and found that similar vertebral body height, shape of the end-plates, shape of the spinal canal, and pedicle size between two species. The size of the superior endplates increased more caudally in the human spine from 27.7 mm of C3 to 54.0 mm of L5, but the size was less increasing in porcine spine, ranging from 20.2 to 28 mm. The same trend was also observed in inferior endplate which indicate the vertebral width in human was larger than porcine. In our study, the screw depth was controlled to 2 thread-widths penetration in bicortical modality and less than 2 mm purchase to opposite cortex in unicortical modality. Although the vertebral width is different between human and porcine, but conditions of insertional depth were controlled in our two modalities. The porcine spine could not be a totally representative model for the human spine, but we believe that for biomechanical studies emphasizing in insertional locations of lateral vertebral body and effect of cortical purchase, the porcine spine still could be an alternative model. To increase the reproducibility and reliability of the pullout data, the screws were pulled out along the same axis as the pullout arm by using a custom-made grip equipped with two universal joints on the upper and lower side of Instron wedge grip, respectively, to achieve coaxial alignment of the screw and the pullout arm^40,41^.

Cage or bone graft subsidence due to screw loosening after anterior spinal instrumentation was reported to range from 3.2 to 7.7% and required additional corrective revision surgery^12,13,15^. Differences in biomechanical comparative results between anterior spinal fixation and posterior pedicle screw stabilization may have been due to variations in the functional spinal unit^18–23^, the insertion position of anterior lateral screws and the difference between unicortical and bicortical fixation approaches. To date, no article has discussed the pullout strength of anterior screws in different positions of the lateral vertebral body or the effect of bicortical fixation.

The values of the decreased pullout force percentage in anterior third of the vertebra were significantly higher than those in the middle and posterior positions (60.7–74% compared to 30–39.5%, respectively), which indicated that screws in a middle or posterior position with bicortical fixation reached better stability.

There were some limitations in our study. First, our data represent only pullout force in a context of normal bone density; osteoporotic bone density should be compared for further clinical applications. Second, porcine models cannot totally represent human vertebrae, and physiological cyclic loading or bending force could be applied for long-term evaluation. Third, only one screw size was examined; further investigations with various sizes and screw geometries might be necessary.

## Conclusion

Our study using porcine vertebrae showed that screws in the middle or posterior position of the lateral vertebral body had a higher pullout performance than those in the anterior position. Posteriorly positioned lateral vertebral screws with unicortical fixation provided better stability than anteriorly positioned screws with bicortical fixation.

## Materials and methods

This study was approved by the committee of Chang Gung Memorial Hospital of Taiwan (CRRPG3M0121). All specimens were purchased from commercial meat market (Yahsen Frozen Foods Co., Taiwan) and were exempted from filing an Institutional Animal Care and Use Committees (IACUCs) protocol for the use of dead animal-derived bone.

### Specimen preparation and screw implantation

A total of 72 fresh frozen L1–6 single vertebrae were used in the study. Specimens were harvested from 12 mature pigs (weight: 80–90 kg). All animals were healthy before harvesting and had never been exposed to any drugs or procedures that could have affected bone density^36,42^. All the specimens were separated into individual vertebrae after being stripped of the surrounding soft tissue. The specimens were conditionally allocated into six groups from L1 to L6 to avoid size-induced selection bias.

**Figure 8:**
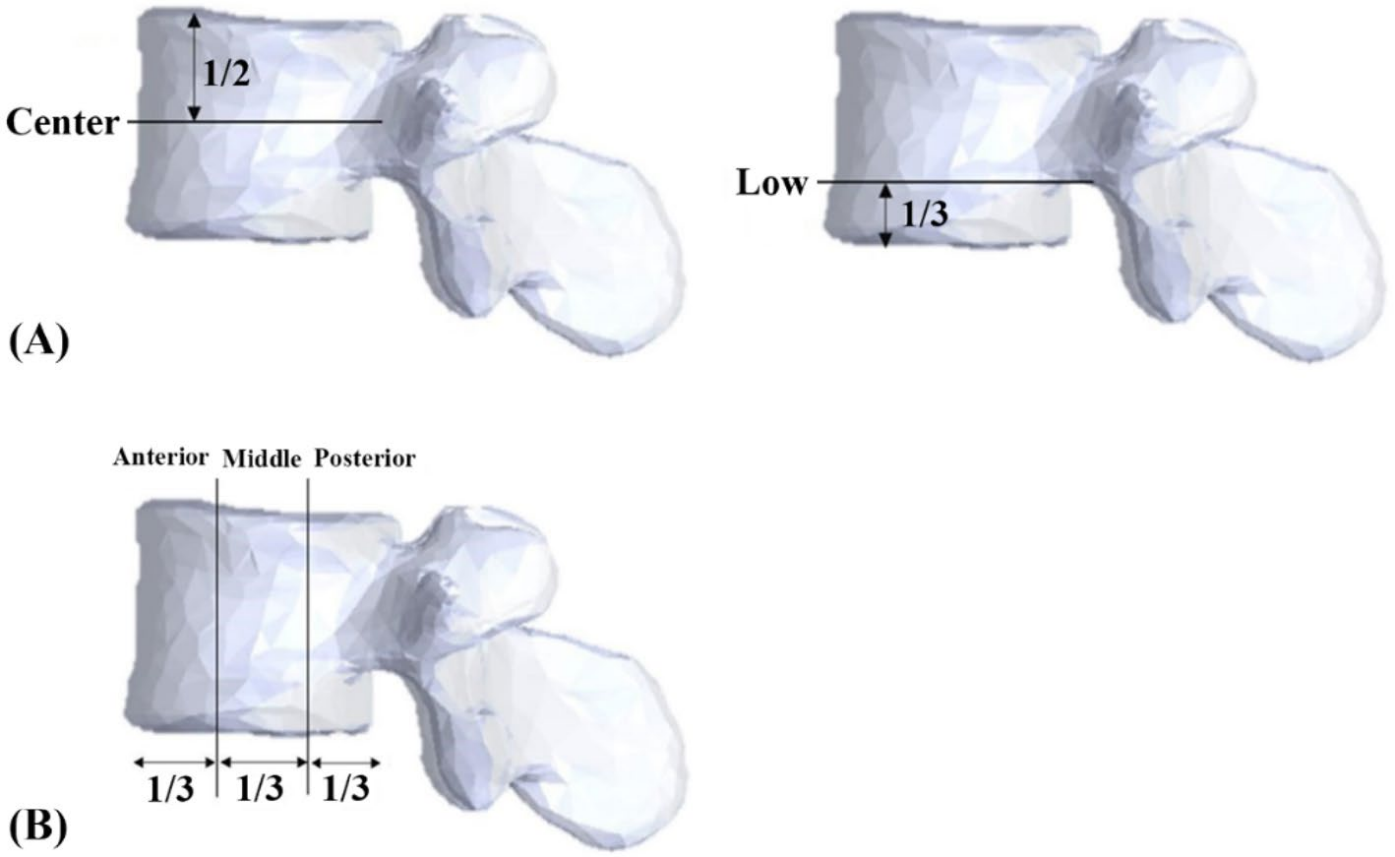
Designation of screw insertion positions. (**A**) The central position is defined as the middle part of the superior and inferior endplates in the lateral plane of the vertebra, while the lower position is the lower third between the superior and inferior endplates. (**B**) The anterior position is defined as the middle of the anterior third of the vertebral body, the middle position is the middle of the middle third and posterior position is the middle of the posterior third.

**Figure 9:**
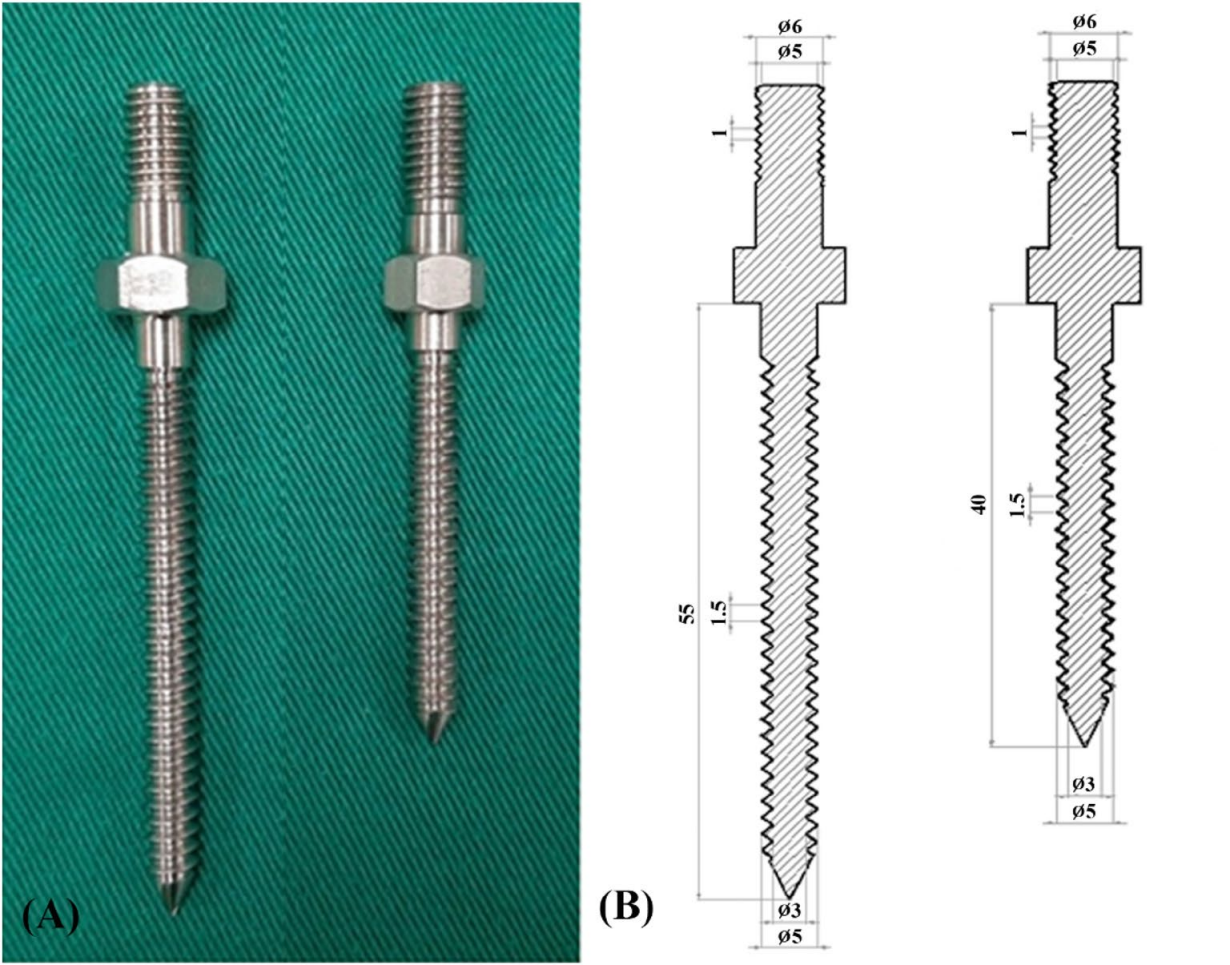
(**A**) Photograph and (**B**) schematic drawing showing the pedicle screws with different lengths. Screws with a dimension (diameter × length) of 5.0 mm × 55 mm were used in the bicortical fixation modality ((**B**), left side) and that of 5.0 mm × 40 mm were used in the unicortical fixation modality ((**B**), right side).

**Figure 10:**
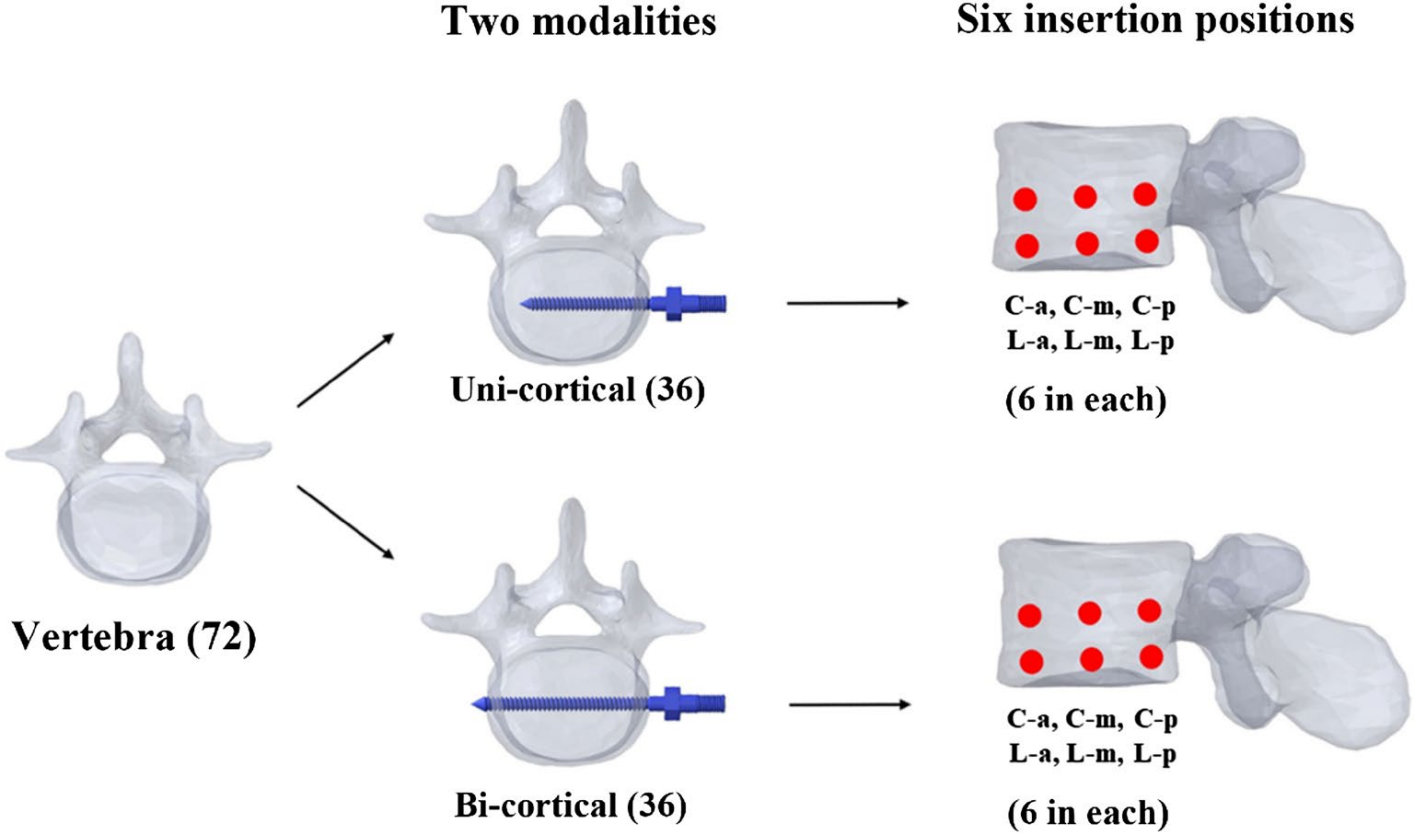
Allocation of the specimens to experimental groups. A total of 72 porcine vertebrae were used. Thirty-six of the 72 porcine vertebrae were randomly designed to the unicortical fixation modality, and the other 36 were randomly designed to the bicortical fixation modality; 6 specimens were used in each group.

All specimens were stored at − 20 °C until the day of testing and thawed for 24 h before implantation. The six groups of screw insertional positions were designated central-anterior (C-a), central-middle (C-m), central-posterior (C-p), lower-anterior (L-a), lower-middle (L-m), and lower-posterior (L-p). The central position was defined as the middle part of the superior and inferior endplates in the lateral plane of vertebra, while the lower position was the lower third between the superior and inferior endplates (Fig. 8A). The anterior position was defined as the middle of the anterior third of the vertebral body, the middle position was the middle of the middle third, and the posterior position was the middle of the posterior third (Fig. 8B). A pilot hole was drilled using a 2.5 mm “twist” metric drill bit attached to a Dremel 4000 rotary tool that was mounted on a Dremel WorkStation Model 220-01. The pilot tract was established parallel to the endplate and posterior vertebral cortex to a depth of 20 mm. Cylindrical screws with diameter × length dimensions of 5.0 mm × 55 mm were used for the bicortical fixation modality, and those with dimensions of 5.0 mm × 40 mm were used for the unicortical fixation modality (Fig. 9). Thirty-six of these seventy-two porcine vertebrae were randomly assigned to the unicortical fixation modality group, and the other 36 were assigned to the bicortical fixation modality group; 6 specimens were used in each group (Fig. 10).

### X-ray image

Axial and sagittal views were examined via X-ray for all specimens prior to the pullout test to confirm an appropriate screw trajectory and insertion depth. All screws were implanted into the vertebrae by an experienced surgeon, and the insertional depth in the unicortical fixation modality was controlled such that there was at least 2 mm from the screw tip to the opposite cortex; in the bicortical fixation modality, at least 2 thread widths penetrated out of the opposite cortex. The vertebrae were thoroughly examined to rule out any malposition or fractures caused by screw insertion.

### Pullout test

**Figure 11:**
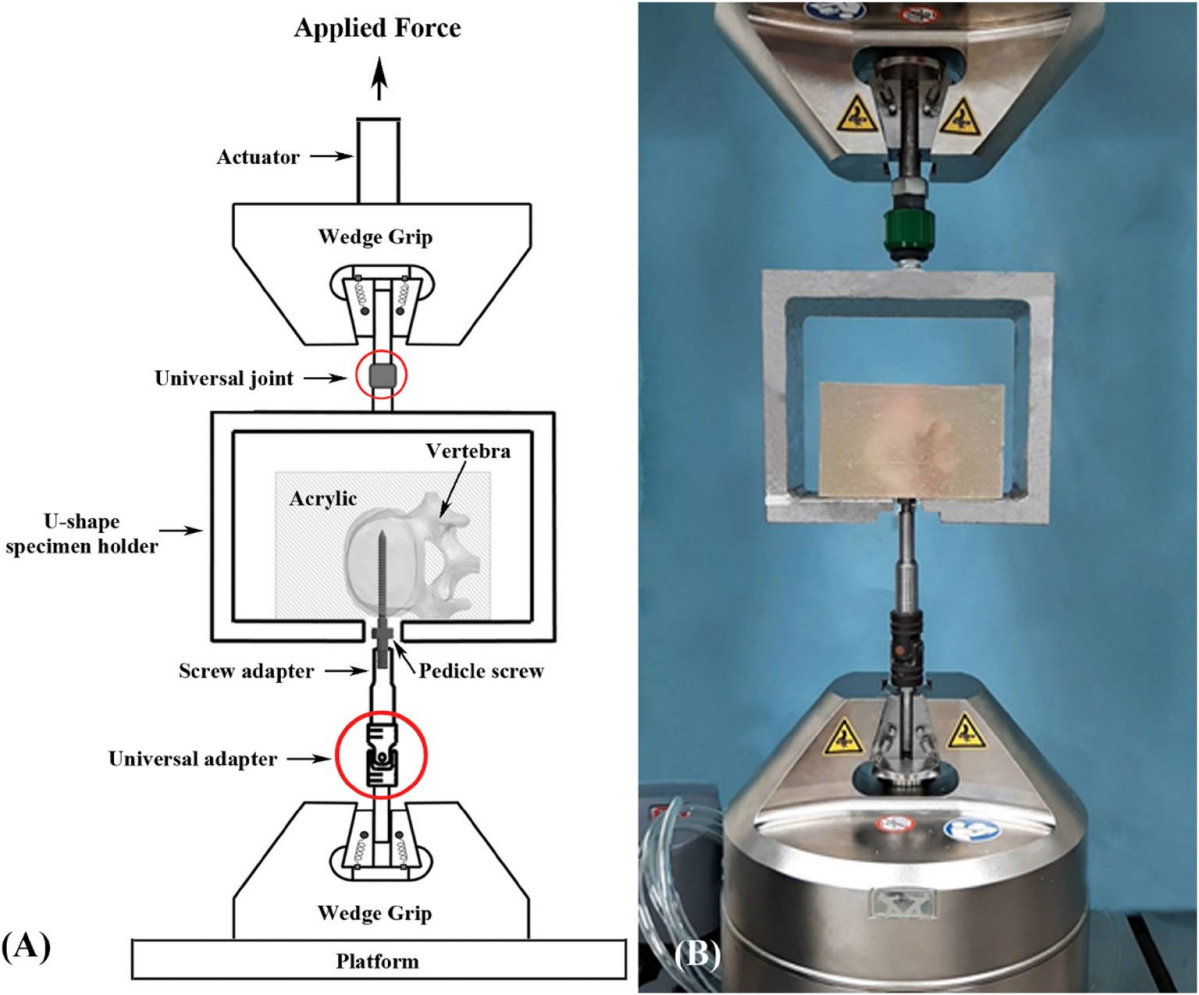
(**A**) Schematic drawing and (**B**) photograph showing the experimental setup of the screw pullout test. Porcine vertebra was potted in specific epoxy resins. The prepared specimens were placed on a specifically designed universal fixture with a self-aligning function and clamped on the upper side of the Instron testing machine to conduct the axial pullout tests.

Each of the 72 specimens was potted in metal boxes using specific epoxy resins (Buehler, Lake Bluff, IL, USA). The specimens were carefully potted into acrylic resin in a cubic shape to fit the square clamp below the universal joint, and the screw head outside the vertebrae was covered with pottery clay to avoid the contact between resin and screw. The prepared specimens were mounted onto a material testing machine (E10000/E10BMTB19359., Instron Com., Norwood MA, USA) to conduct axial pullout tests with the screws. The screw head was fixed to a cylindrical rod with an inner thread that matched the outer thread of the screw head via a universal adaptor (Fig. 11). The universal adaptor was then clamped to the lower wedge grip of the testing machine. The potted specimen was secured on a custom-made upper grip capable of universal rotation to achieve the coaxial alignment of the screw with the pullout arm. We ensured that the screws and the pullout force were directed along the same axis by clamping the potted specimen in a way that enabled free rotation about the axis, and the direction of the pullout force could be adjusted through the polyaxial design of the adaptor. The screws were then loaded in displacement control mode at a constant displacement rate of 5 mm/min for a total displacement of 10 mm, which is in accordance with published literature on axial pull-out testing^42–44^. Data collection was set at 1 sample/0.05 mm. Failure was defined as the maximum force or the force peak prior to a decrease in force associated with increasing displacement. After the pullout test was completed, the specimen and the screws were closely examined for signs of fracture and damage and any findings were carefully recorded.

### Statistics

Statistical software (SPSS for Windows version 12.0, SPSS Inc., Chicago, IL) was used to analyze the pullout force of all the specimens. All of the measurements were collected for 72 vertebrae and the results were expressed as the mean ± standard deviation (SD). The Kruskal–Wallis test was used to determine significance differences between median values of subgroups. p values were 2-sided and adjusted by Bonferroni correction when multiple comparisons were performed. Mann–Whitney U tests was used to compare unicortical purchased screws in the posterior and middle position to bicortical purchased screws in anterior position. Differences were considered statistically significant at p < 0.05.

## Data Availability

All data generated or analyzed during this study are included in this published article.
